# The p21 levels have the potential to be a monitoring marker for ribociclib in breast cancer

**DOI:** 10.18632/oncotarget.27127

**Published:** 2019-08-06

**Authors:** Masafumi Iida, Misato Nakamura, Emi Tokuda, Daichi Toyosawa, Toshifumi Niwa, Noriaki Ohuchi, Takanori Ishida, Shin-ichi Hayashi

**Affiliations:** ^1^Department of Molecular and Functional Dynamics, Graduate School of Medicine, Tohoku University, Sendai, Japan; ^2^Department of Breast and Endocrine Surgical Oncology, Graduate School of Medicine, Tohoku University, Sendai, Japan

**Keywords:** breast cancer, CDK4/6 inhibitor, resistance, CDK6, p21

## Abstract

Although cyclin-dependent kinase (CDK) 4/6 inhibitors have exhibited remarkable results for patients with estrogen receptor (ER)–positive breast cancer in clinical trials, the mechanism of CDK4/6 inhibitor resistance remains unclear. Thus, this study aimed to investigate the mechanism of CDK4/6 inhibitor resistance using two CDK4/6 inhibitor resistant breast cancer cell lines. We established CDK6 overexpressed cell lines (MCF7-C6) from MCF-7 cells using the stably transfected CDK6 expression vector. Additionally, acquired ribociclib-resistant (RIBR) cell lines were created using ER-positive hormone-resistant cell lines by long-term exposure to ribociclib. CDK6 overexpression and the knockdown of CDK4 experiments highlight the significance of high levels of CDK4 and low levels of CDK6 in CDK4/6 inhibitor sensitivity. Moreover, RIBR cell lines did not exhibit incremental CDK6 compared with ER-positive hormone-resistant cell lines. In MCF7-C6 and RIBR cell lines, p21 levels decreased, and p21 levels were proportional to CDK4/6 inhibitor sensitivity. This study suggests that overexpression of CDK6 is one of the many possible mechanisms of resistance to CDK4/6 inhibitors. Furthermore, p21 levels have the potential to serve as a marker for CDK4/6 inhibitors independent of the resistance mechanism.

## INTRODUCTION

Until recently, the primary therapeutic options for postmenopausal women with estrogen receptor (ER)–positive human epidermal growth factor receptor 2 (HER2)-negative metastatic breast cancer (MBC) were predominantly comprised of hormonal therapy [1, 2]. Although hormonal therapy is a markedly effective treatment for patients with ER-positive breast cancer with few side effects, some types of breast cancer acquire resistance over time, leading to relapse during or after hormonal therapy. In previous studies, we researched the mechanism of aromatase inhibitor (AI) resistance by establishing multiple AI-resistant breast cancer cell line models and reporting on several mechanisms of AI resistance [3–6]. Our research elucidated that the effective therapy for hormone resistant models differs based on the resistance mechanism. Clinically, hormonal therapy resistance is considered a critical problem and is correlated with the involvement of intracellular phosphorylation pathways, such as the PI3K–AKT–mTOR pathway and MAPK pathways [7, 8]. Previously, we reported that driver signaling escapes to MAPK signaling despite the suppression of mTOR signaling [9]. Because of this, breast cancer cells struggle to survive by altering the driver pathway. Targeting downstream signaling of ER and intracellular phosphorylation pathways (i.e. the cell cycle) is a logical, rational strategy for breast cancer drug therapy.

In recent years, cyclin-dependent kinase (CDK)4/6 inhibitors have been developed as a revolutionary class of novel drugs, exhibiting excellent outcomes in clinical trials, which resulted in their approval and clinical use worldwide [10–12]. Primarily, three CDK4/6 inhibitors—palbociclib, ribociclib, and abemaciclib—have been developed [13], all of which will likely be used extensively in clinical settings. However, we are still confronted with several clinically relevant questions about CDK4/6 inhibitors, such as the treatment regimen, detection of biomarkers, resistance mechanism, and treatment after CDK4/6 inhibitor-resistance.

CDK families are essential in the cell-cycle regulation, making them a therapeutic target to interfere with the cell-cycle division and proliferation. A complex of CDK4 and CDK6 with cyclin D results in the activation of CDK4/6, which initiates the phosphorylation and inactivation of retinoblastoma (Rb) protein, resulting in immigration to the S-phase of the cell cycle [14–16]. CDK4/6 inhibitors are predominately used in the treatment of ER-positive breast cancer, as the aberration of the cyclin D1–Rb pathway has been reported more frequently in ER-positive breast cancer [17]. In addition, preclinical data have determined a luminal subtype, elevated expression of cyclin D1 and Rb protein, and decreased p16 expression [18]. Despite the fact that these factors act as biomarkers for CDK4/6 inhibitors, they remain inadequate tools to predict sensitivity to CDK4/6 inhibitors [19].

In this study, we established acquired CDK4/6 inhibitor–resistant cell lines from hormone-resistant cell lines with positive ER expression. Almost all patients with ER-positive breast cancer were administered antihormonal drugs as adjuvant therapy. In addition, a CDK4/6 inhibitor was prescribed in combination with antihormonal drugs in cases of MBC. Hence, the addition of AI resistance to CDK4/6 inhibitor resistance is a plausible method for elucidating the resistance mechanism and assessing effective therapeutic options following resistance to CDK4/6 inhibitor, although CDK4/6 inhibitors are used as first-line therapy of MBC.

This study aimed to assess the impact of CDK4 and CDK6 expression levels on the CDK4/6 inhibitor sensitivity. In addition, we intended to establish ribociclib-resistant cell lines from estrogen deprivation–resistant (EDR) 1 as ER-positive hormone-resistant cell lines, elucidate the mechanism of CDK4/6 inhibitor resistance, and investigate the effective therapeutic options following CDK4/6 inhibitors resistance.

## RESULTS

### Correlation between the efficacy of CDK4/6 inhibitor and CDK4 and CDK6 expression levels

**Figure 1:**
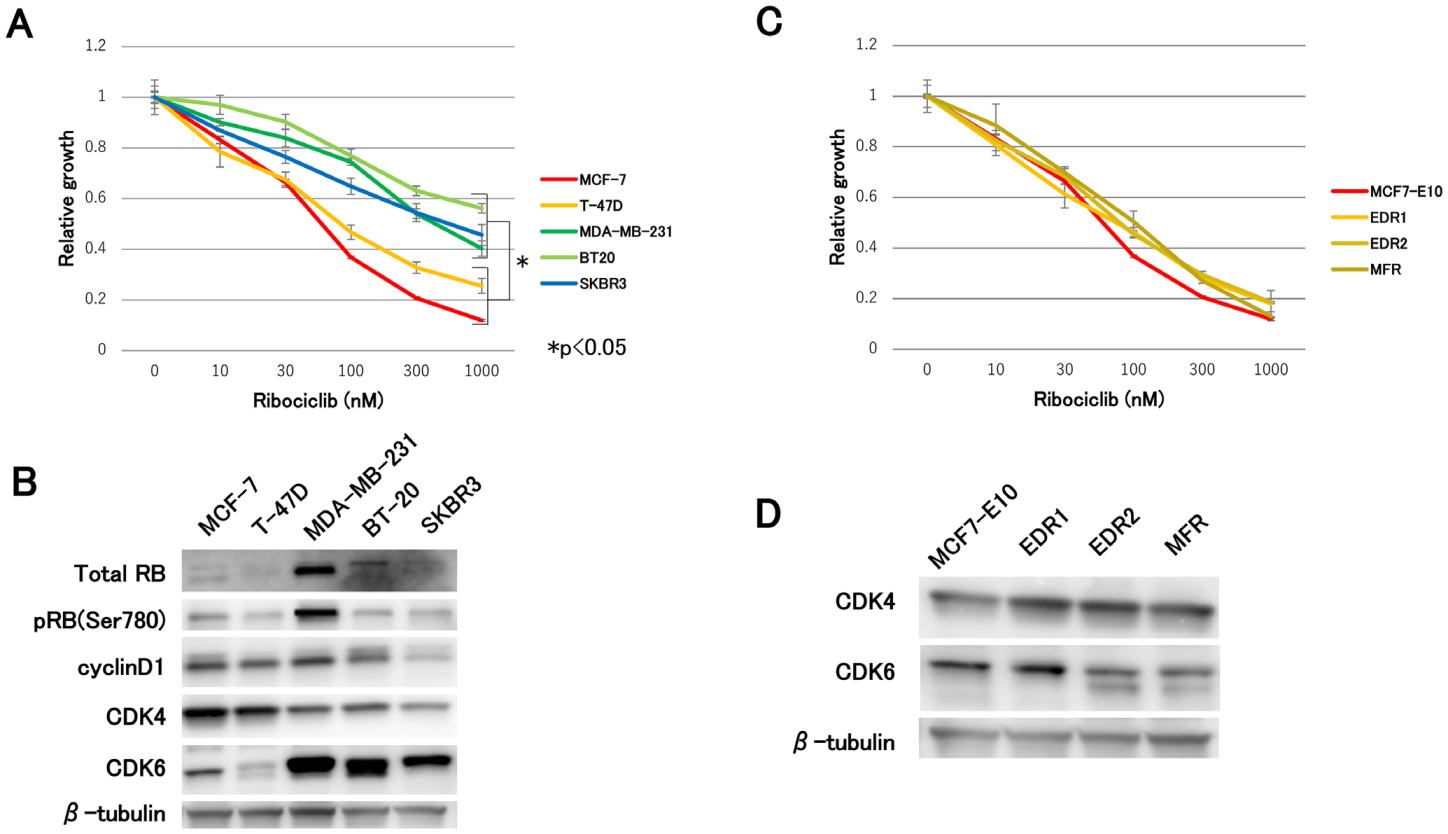
Effect of ribociclib in breast cancer cell lines and hormone-resistant cell lines. (**A**) Cell proliferation assay of each subtype of breast cancer cell lines treated with ribociclib for 3 days was measured relative to the negative control (treated with DMSO). The results are presented as mean ± standard deviation (SD), and *P* < 0.05 was considered significant. (**B**) Protein expression levels of cell-cycle regulatory agents were analyzed using western blotting, with β-tubulin as a protein loading control. (**C**) Cell proliferation of hormone-resistant breast cancer cell lines treated with ribociclib for 3 days was measured relative to the negative control. (**D**) Protein expression levels of CDK4 and CDK6 were analyzed by western blotting with β-tubulin as a protein loading control.

First, we assessed the efficacy of ribociclib for each subtype of breast cancer cell lines. Compared with non-luminal-type cell lines (MDA-MB-231, BT20, and SKBR3), luminal-type cell lines (MCF-7 and T-47D cells) suppressed cell growth to a large extent (Figure 1A). The most dramatic difference between these two groups was observed in the CDK4 and CDK6 expression levels. Specifically, immunoblot analysis of luminal-type cell lines exhibited lower levels of CDK6 and higher levels of CDK4, while non-luminal-type cell lines exhibited the opposite (Figure 1B). In addition, we investigated the efficacy of ribociclib in hormone-resistant cell lines, which were previously established in our laboratory. After long-term estrogen depletion, two clones of AI-resistant breast cancer models were derived from MCF7-E10 cell lines. While EDR1 displayed elevated ER expression levels, EDR2 displayed decreased ER expression levels [3]. We obtained fulvestrant-resistant (MFR) cell lines derived from MCF7-E10 cell lines that had completely lost ER expression [20]. No remarkable differences were observed in ribociclib sensitivity between MCF7-E10 and these hormone-resistant cell lines (Figure 1C). Regardless of the mechanism of hormonal therapy resistance, ribociclib was effective against hormone-resistant cell lines. The CDK4 and CDK6 expression levels presented were comparable to MCF-7 in this study (Figure 1D). These findings suggested a possible correlation between the ribociclib sensitivity and the CDK4 and CDK6 expression levels.

### Resistance to CDK4/6 inhibitor due to the CDK6 overexpression

**Figure 2:**
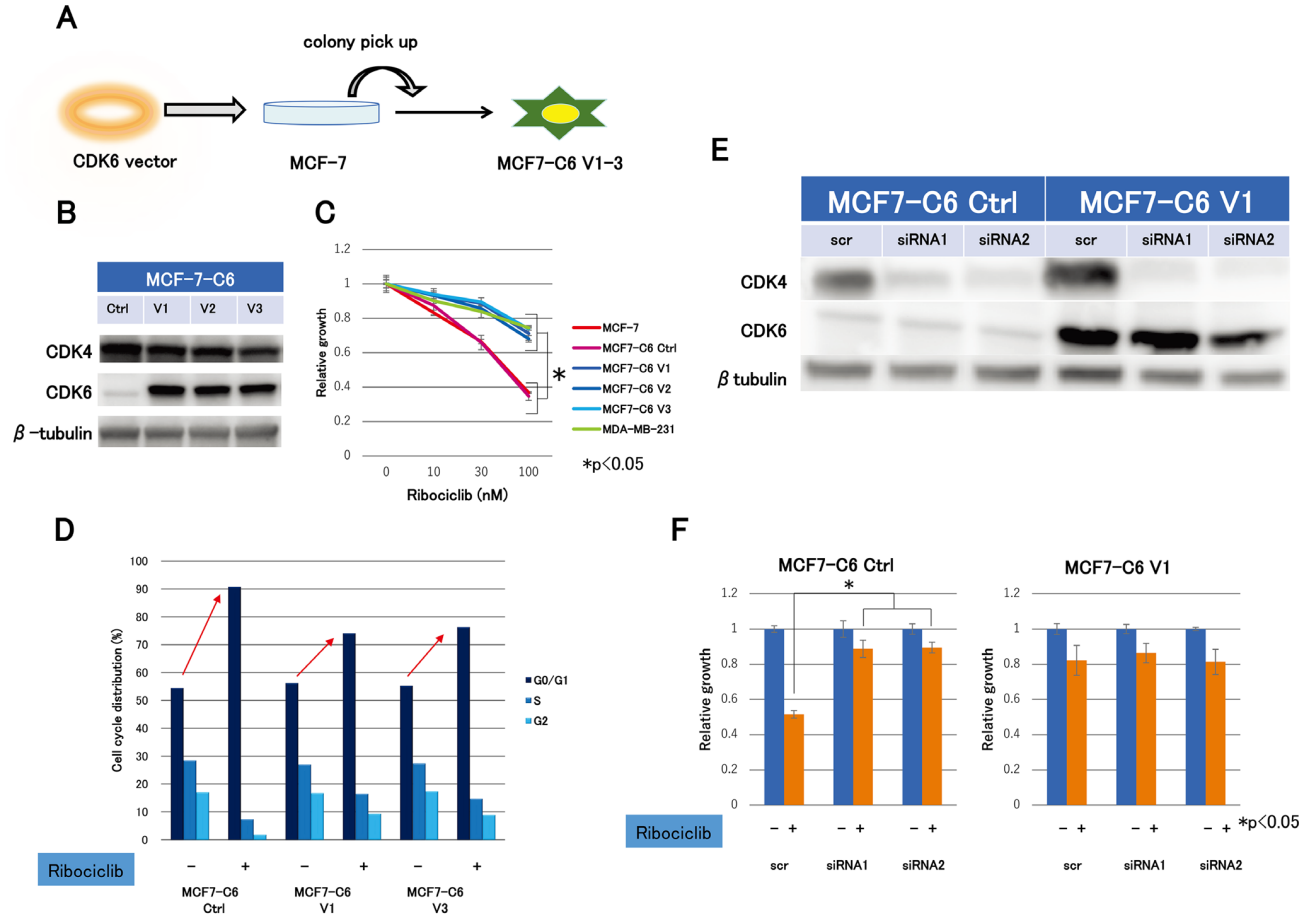
Expression levels of CDK4- and CDK6-producing effect to ribociclib sensitivity. (**A**) CDK6 overexpression cell lines (MCF7-C6) were established from MCF-7 by the stably transfected CDK6 expression vector. Cell lines–transfected control vector (MCF7-C6 Ctrl) was simultaneously established as a negative control. (**B**) Western blotting demonstrated the protein expression levels of CDK4 and CDK6 in MCF7-C6 Ctrl and MCF7-C6 cell lines. (**C**) Cell proliferation assay of MCF-7, MCF7-C6 Ctrl, MCF7-C6, and MDA-MB-231 cell lines treated with ribociclib for 3 days was measured relative to the negative control (treated with DMSO). The results are expressed as mean ± SD of three independent experiments; ^*^ *P* < 0.05. (**D**), MCF7-C6 Ctrl and MCF7-C6 cell lines were treated with DMSO or ribociclib (500 nM) for 24 h and measured by fluorescence-activated cell sorting (FACS) cell-cycle analysis. (**E**), two kinds of siRNA for CDK4 (siRNA1, siRNA2) or nonspecific control siRNA (scr) were transfected for 24 h with MCF7-C6 Ctrl and MCF7-C6 V1 cell lines. Protein expression levels of CDK4 and CDK6 were analyzed by western blotting with β-tubulin as a protein loading control. (**F**) Cell proliferation of MCF7-C6 Ctrl -scr, -siRNA1, -siRNA2, MCF7-C6 V1-scr, -siRNA1, and -siRNA2 treated with ribociclib (100 nM) for 3 days was measured relative to dishes treated with DMSO as a negative control. The results are expressed as mean ± SD of two independent experiments; ^*^ *P* < 0.05.

In this study, we established three clones of CDK6 overexpressed cell lines (MCF7-C6 V1-3) from MCF-7 by stably incorporating the transfected CDK6 expression vector in order to explore the molecular mechanism of the ribociclib sensitivity (Figure 2A). Concurrently, we established MCF7-C6 control cell lines (MCF7-C6 Ctrl) using the transfected control vector as a negative control. Immunoblot analysis revealed that CDK6 was indeed transfected and highly expressed (Figure 2B). Remarkably, MCF7-C6 cell lines were less sensitive to ribociclib than MCF7-C6 Ctrl cell lines, which were consistent with MDA-MB-231 (Figure 2C). In addition, cell cycle analysis revealed that G1 arrest by ribociclib in MCF7-C6 cell lines was markedly reduced compared with MCF7-C6 Ctrl cell lines (Figure 2D). These results suggested that the CDK6 overexpression accounts for ribociclib resistance.

### High levels of CDK4 and low levels of CDK6: a possible key factor for the efficacy of CDK4/6 inhibitors

We performed CDK4 knockdown experiments using CDK4 siRNA in MCF7-C6 Ctrl and MCF7-C6 V1 cell lines to further investigate the molecular mechanism of ribociclib sensitivity. With the CDK4 knockdown, we created cells with four different expression patterns of CDK4 and CDK6 (Figure 2E). Ribociclib sensitivity was extremely high only in cells expressing high CDK4 and low CDK6 compared to cells with other expression patterns (Figure 2F), suggesting CDK4 expression levels are a crucial factor for ribociclib sensitivity. These findings revealed that CDK4/6 inhibitor sensitivity could be associated with both high levels of CDK4 and low levels of CDK6.

### Establishment of acquired resistant cell lines for ribociclib

**Figure 3:**
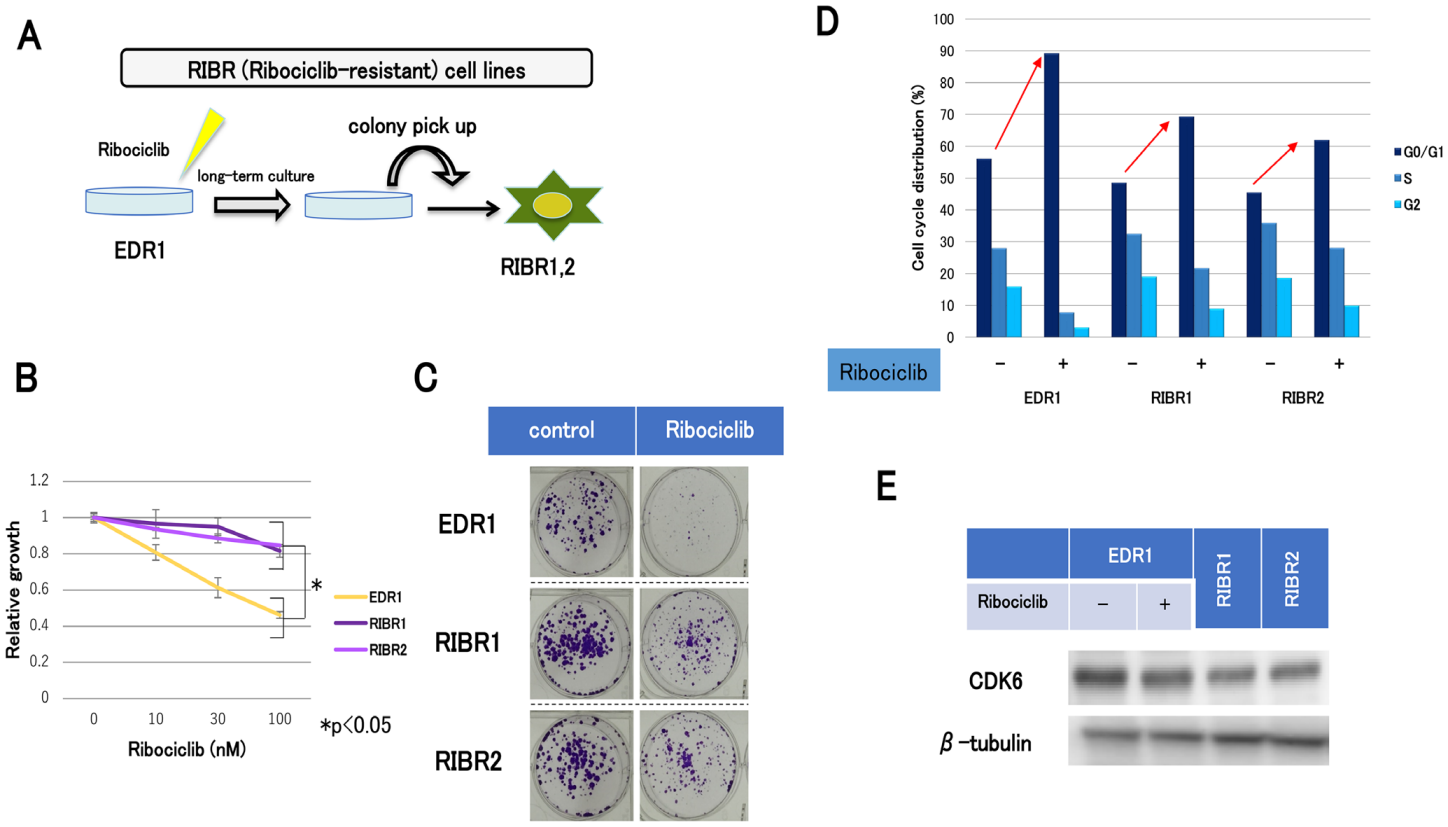
The establishment of ribociclib-resistant cell lines (RIBR). (**A**) EDR1 was established from MCF7-E10 cell lines by long-term culture with a steroid-depleted medium. MCF7-E10 cell lines were derived from MCF-7, which had been stably transfected with an ERE-GFP reporter plasmid, and monitored ER expression. EDR1 exhibited ER overexpression. RIBR was established from EDR1 after long-term (7 months) culturing with 1000 nM ribociclib in phenol red–free RPMI medium. (**B**) Cell proliferation of EDR1 and RIBR1,2 treated with ribociclib for 3 days was measured relative to the negative control. The results are expressed as mean ± SD of three independent experiments; ^*^ *P* < 0.05. (**C**) Colony formation assay of EDR1 and RIBR1,2 cell lines. The control (left) was cultured for 15 days without any drugs. Ribociclib (right) was harvested for 15 days with 1000 nM ribociclib. (**D**), EDR1 and RIBR1,2 cell lines were treated with DMSO or ribociclib (1000 nM) for 24 h and measured by the fluorescence-activated cell sorting (FACS) cell-cycle analysis. (**E**), the protein expression levels of CDK6 in EDR1, EDR1 with 1000 nM ribociclib for 24 h, and RIBR1,2 cell lines were analyzed by western blotting.

After long-term culture under conditions of sufficient doses of ribociclib, we established acquired resistant cell lines for ribociclib (RIBR1, 2) from EDR1 (Figure 3A). RIBR cell lines were less sensitive to ribociclib than EDR1 (Figure 3B). In addition, colony-forming abilities of RIBR cell lines in the presence of ribociclib were higher compared with those in the presence of EDR1 (Figure 3C). Cell-cycle analysis suggested that G1 arrest by ribociclib in RIBR cell lines was reduced compared with parent cells (Figure 3D). Remarkably, CDK6 expression levels were not elevated in RIBR cell lines (Figure 3E), suggesting that the mechanism of ribociclib resistance is different between MCF7-C6 and RIBR cell lines.

### Cross-resistance to other CDK4/6 inhibitors

**Figure 4:**
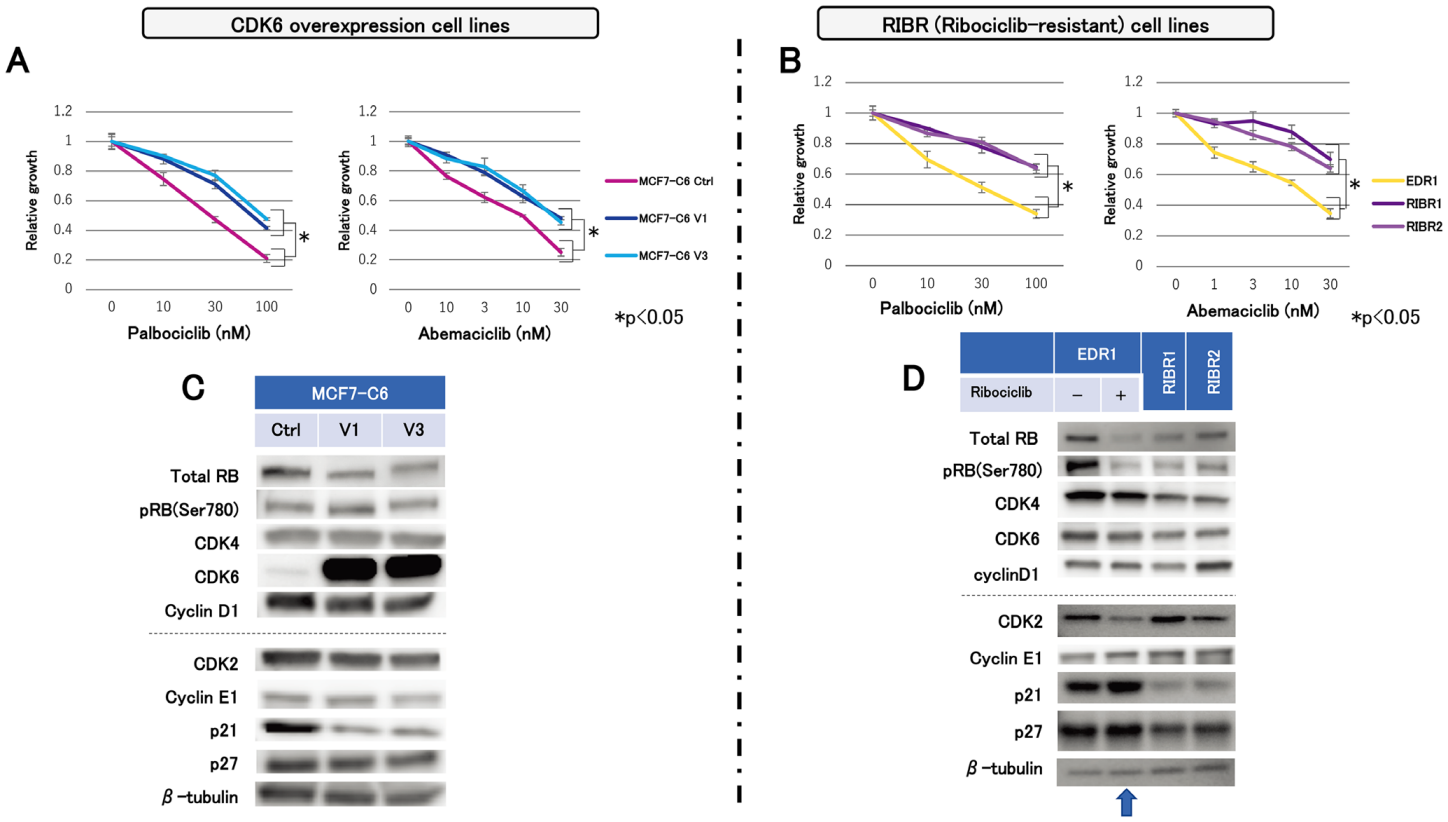
Sensitivity to other CDK4/6 inhibitors and molecular-targeted drugs and expression levels of cell-cycle regulatory agents. Cell proliferation of MCF7-C6 (**A**) and RIBR (**B**) cell lines treated with palbociclib and abemaciclib for 3 days was measured relative to the negative control. The results are expressed as mean ± SD of three independent experiments; ^*^ *P* < 0.05. (**C**) Protein expression levels of cell-cycle regulatory agents in MCF7-C6 and (**D**) EDR1, EDR1 with 1000 nM ribociclib for 24 h (indicated by an arrow) and RIBR1,2 cell lines were analyzed using western blotting, with β-tubulin as a protein loading control.

We assessed the efficacy of other CDK4/6 inhibitors on MCF7-C6 and RIBR cell lines, which have different resistant mechanisms. MCF7-C6 cell lines displayed lower sensitivity to palbociclib than MCF7-C6 Ctrl cell lines (Figure 4A). In addition, RIBR cell lines expressed lower sensitivity to palbociclib than EDR1 (Figure 4B). Furthermore, MCF7-C6 and RIBR cell lines exhibited the decreased abemaciclib sensitivity than each of the control cells (Figure 4A and 4B), suggesting that MCF7-C6 and RIBR cell lines acquired cross-resistance to not only palbociclib but also abemaciclib.

### Decline in p21 levels after resistance to CDK4/6 inhibitor

In MCF7-C6 and RIBR cell lines, we assessed the protein levels of cell-cycle regulatory agents by immunoblot analysis. We focused on the CDK4 expression levels and its related factors at first because the CDK4:CDK6 ratio might be associated with the CDK4/6 inhibitor upon investigation. We observed no remarkable changes in the total Rb, CDK4, and cyclin D1 between MCF7-C6 and MCF7-C6 Ctrl cell lines (Figure 4C). In contrast, RIBR cell lines expressed lower levels of total RB, pRB(Ser780), and CDK4 than EDR1 (Figure 4D). Subsequently, we investigated CDK2 expression levels and its related factors. Interestingly, p21 expression levels were markedly reduced in both MCF7-C6 and RIBR cell lines, while CDK2 and cyclin E1 expression remained almost unchanged. Of note, these changes in RIBR cell lines were not observed during short-term (24-h) exposure of ribociclib to EDR1 (Figure 4D, indicated by an arrow). These findings revealed that MCF7-C6 and RIBR cell lines expressed low levels of p21, although the resistance mechanisms to CDK4/6 inhibitors were different.

### Efficacy of molecular-targeted and chemotherapeutic drugs

**Figure 5:**
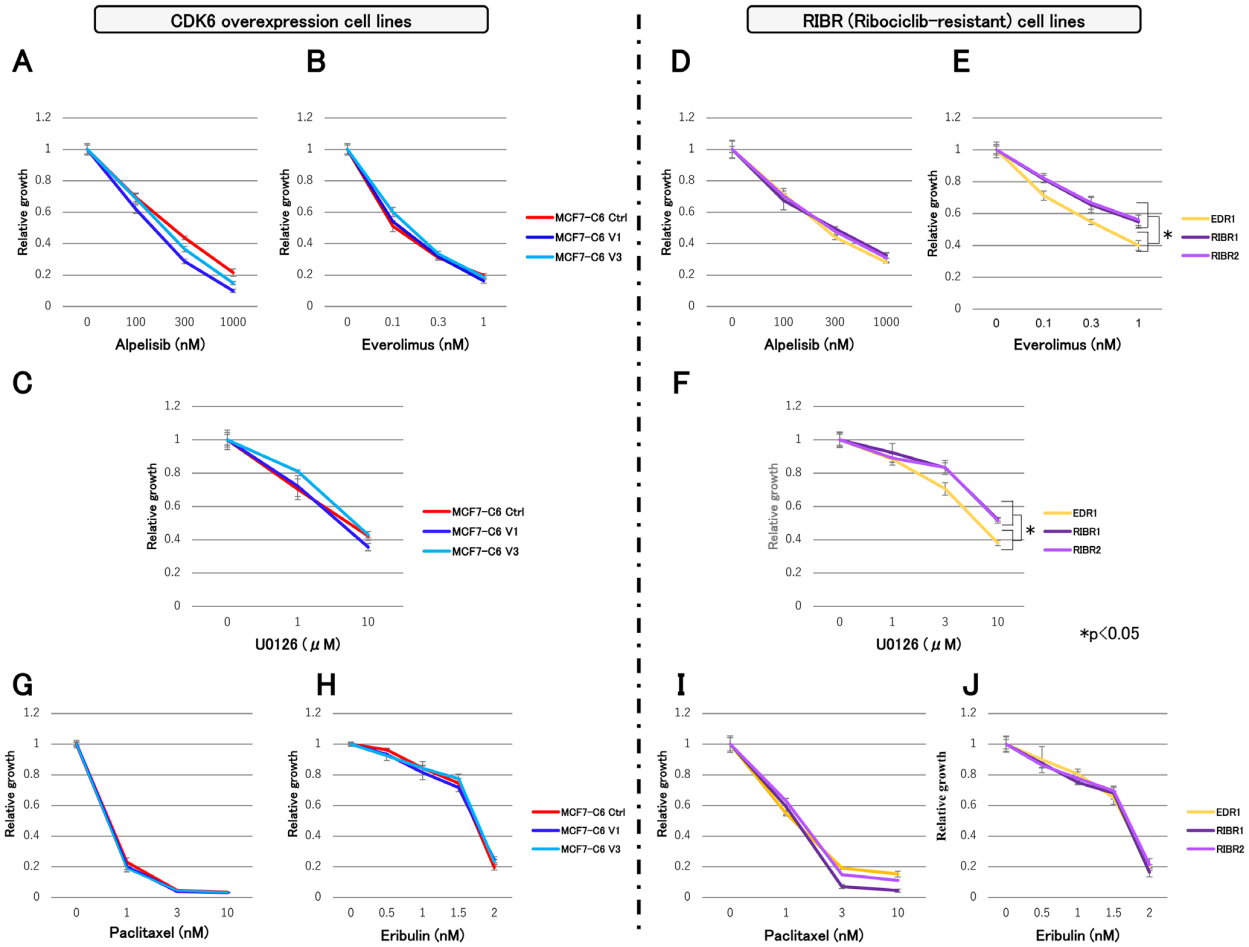
Cell proliferation assay of MCF7-C6 and RIBR cell lines with molecular-targeted and chemotherapeutic drugs. (**A**) MCF-7 C6 cell lines treated with alpelisib (**B**) everolimus, and (**C**) U0126. (**D**) RIBR cell lines treated with alpelisib, (**E**) everolimus, and (**F**) U0126. (**G**) MCF-7 C6 cell lines treated with paclitaxel and (**H**) eribulin. (**I**), RIBR cell lines treated with paclitaxel and (**J**) eribulin. All experiments were treated with each drug for 3 days and measured relative to the negative control. The results are expressed as mean ± SD; ^*^ *P* < 0.05.

We investigated the efficacy of molecular-targeted and chemotherapeutic drugs in MCF7-C6 and RIBR cell lines using the following: alpelisib; α-subunit-specific PI3K inhibitor, everolimus; mTOR inhibitor and U0126; MEK inhibitor. In addition, MCF7-C6 cell lines exhibited suppressed cell growth by these three molecular-targeted drugs equivalent to MCF7-C6 Ctrl cell lines (Figure 5A–5C). While alpelisib sensitivity in RIBR cell lines was equivalent to EDR1, everolimus and U0126 sensitivities were slightly lower than EDR1 (Figure 5D–5F). Furthermore, chemotherapeutic drugs, such as paclitaxel and eribulin, exhibited efficacy in both MCF7-C6 and RIBR cell lines (Figure 5G–5J).

### Restoration of the sensitivity to CDK4/6 inhibitors after long-term ribociclib depletion

**Figure 6:**
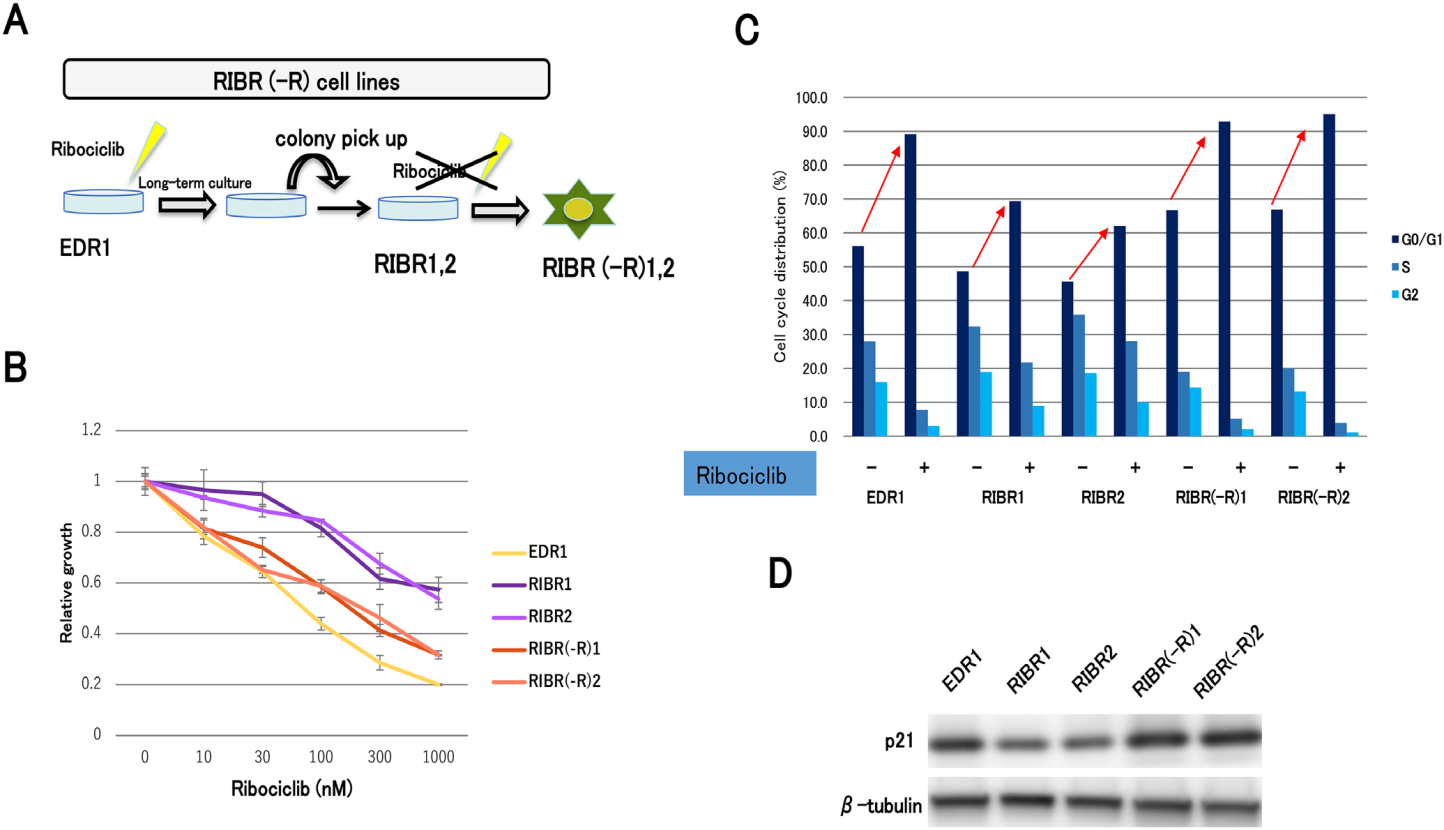
Establishment of ribociclib-resistant cell lines with long-term ribociclib depletion (RIBR(-R)). (**A**) RIBR(-R) cell lines were established from RIBR cell lines by long-term culture (4 months) without ribociclib in the phenol red–free RPMI medium. (**B**) Cell proliferation of EDR1, RIBR1,2, and RIBR(-R)1,2 cell lines treated with ribociclib for 3 days was measured relative to the negative control. The results are expressed as mean ± SD of three independent experiments; ^*^ *P* < 0.05. (**C**) EDR1, RIBR1,2, and RIBR(-R)1,2 cell lines were treated with DMSO or ribociclib (1000 nM) for 24 h and measured by the fluorescence-activated cell sorting (FACS) cell-cycle analysis. (**D**) Protein expression levels of p21 in EDR1, RIBR1,2, and RIBR(-R)1,2 cell lines were analyzed by western blotting.

Finally, we assessed the efficacy of long-term ribociclib depletion to RIBR cell lines. We established RIBR(-R) cell lines from RIBR cell lines by long-term culturing without ribociclib (Figure 6A). We obtained RIBR(-R)1 and RIBR(-R)2 cell lines from RIBR1 and RIBR2 cell lines, respectively. Remarkably, RIBR(-R) cell lines suppressed cell growth to a larger extent than RIBR cell lines (Figure 6B). In addition, cell-cycle analysis revealed G1 arrest by ribociclib in RIBR(-R) cell lines was increased compared with RIBR cell lines (Figure 6C). Interestingly, p21 expression levels in RIBR(-R) cell lines were restored to the same degree as EDR1 (Figure 6D). Considering the results of Figure 4C and 4D, p21 could be associated with the mechanism of CDK4/6 inhibitor resistance.

## DISCUSSION

At present, CDK4/6 inhibitors are promising given the outcomes observed in clinical trials [10–12]. Despite being confronted with several clinical questions about CDK4/6 inhibitors, such as the treatment regimen, detection of biomarkers, resistance mechanism, and the treatment after CDK4/6 inhibitor resistance, patients are likely to reap profound benefits from the use of CDK4/6 inhibitors. In this study, we investigated the mechanism of CDK4/6 inhibitor resistance and variation after CDK4/6 inhibitor resistance using breast cancer cell lines, hormone-resistant cell lines, and two different kinds of CDK4/6 inhibitor resistance cell lines.

The study illustrated that ribociclib sensitivity varied between luminal and non-luminal cell lines. In addition, ribociclib was found to be effective in all hormone-resistant cell lines similar to MCF7-E10 cell lines independent of the mechanism of hormonal therapy resistance (Figure 1A and 1C). The efficacy to CDK4/6 inhibitors is variable, depending on the breast cancer cell line [18]. Although differences were primarily reported as elevated pRb and cyclin D1 and depressed CDKN2A(p16), this study focused on CDK4 and CDK6 expression levels. Cell lines sensitive to ribociclib shared characteristics with high levels of CDK4 and low levels of CDK6, indicating that ribociclib sensitivity could be contributing to CDK4 and CDK6 expression levels. Characterization experiments in CDK6 overexpression cell lines elucidated that CDK6 overexpression resulted in ribociclib resistance. In addition, CDK4 knockdown experiments demonstrated that ribociclib resulted in cell growth inhibition only in cells expressing high levels of CDK4 and low levels of CDK6 (Figure 2F). Perhaps, the prediction of the CDK4/6 inhibitor sensitivity could determine the levels of CDK4 and CDK6 in breast cancer. In other words, CDK4 and CDK6 levels could potentially become a biomarker for CDK4/6 inhibitors.

Acquired CDK6 amplification has resulted in breast cancer resistance to CDK4/6 inhibitors [21]; however, the reason for CDK6 overexpression resulting in resistance to CDK4/6 inhibitors remains unclear. One study reported a correlation between the presence of active CDK4 and the palbociclib sensitivity in breast cancer cell lines and their tumor models [22], implying that CDK4/6 functions could not be identical. Although the precise functions of CDK4/6 remain partially understood, both CDK4/6, particularly CDK6, may have an entirely different function, which promotes cell growth.

In contrast, the RIBR model of acquired resistance to CDK4/6 inhibitors exhibited CDK6 expression levels similar to EDR1. Although CDK4 expression levels in RIBR cell lines were marginally decreased compared to EDR1 (Figure 4D), a mild reduction could be attributed to the process of acquired resistance but not fundamental causes. A difference in CDK6 expression levels between MCF7-C6 and RIBR cell lines suggested diverse mechanisms of resistance. The modestly decreased RB and pRB levels in RIBR cell lines compared to those in EDR1 could be the major resistance mechanism in RIBR cell lines. Some mechanisms of CDK4/6 inhibitor resistance have been previously reported, including CDK6 amplification [21], loss of Rb or CCNE1 amplification [23] and CDK2 activity [24]. Breast cancer cell lines characterized by loss of RB1 activity are hyposensitive to CDK4/6 inhibitors; however, loss of RB1 function is rare in ER-positive breast cancer [25, 26]. RB1 mutations occurring during acquired resistance to palbociclib were found in the palbociclib group but not the placebo group in Paloma-3 trial [27]. Given these data, loss of RB1 function is considered a common cause of CDK4/6 inhibitor resistance, but this does not explain the entirety CDK4/6 inhibitor resistance mechanisms because the number of RB1 mutations in the Paloma-3 trial was only around 5%. The present study determined the presence of multiple mechanisms in the resistance of CDK4/6 inhibitors. Remarkably, we observed similarities between MCF7-C6 and RIBR cell lines i.e., declined p21 expression levels (Figure 4C and 4D). In addition, RIBR(-R) cell lines restored p21 expression levels (Figure 6D). p21 expression is strictly regulated by p53 [28]. In this study, we assessed p53 expression; however, expression levels were quite low and remained almost unchanged in these cell lines (data not shown). Moreover, RIBR(-R) cell lines restored sensitivity to CDK4/6 inhibitors (Figure 6B), suggesting that sensitivity to CDK4/6 inhibitors is reversible.

**Figure 7:**
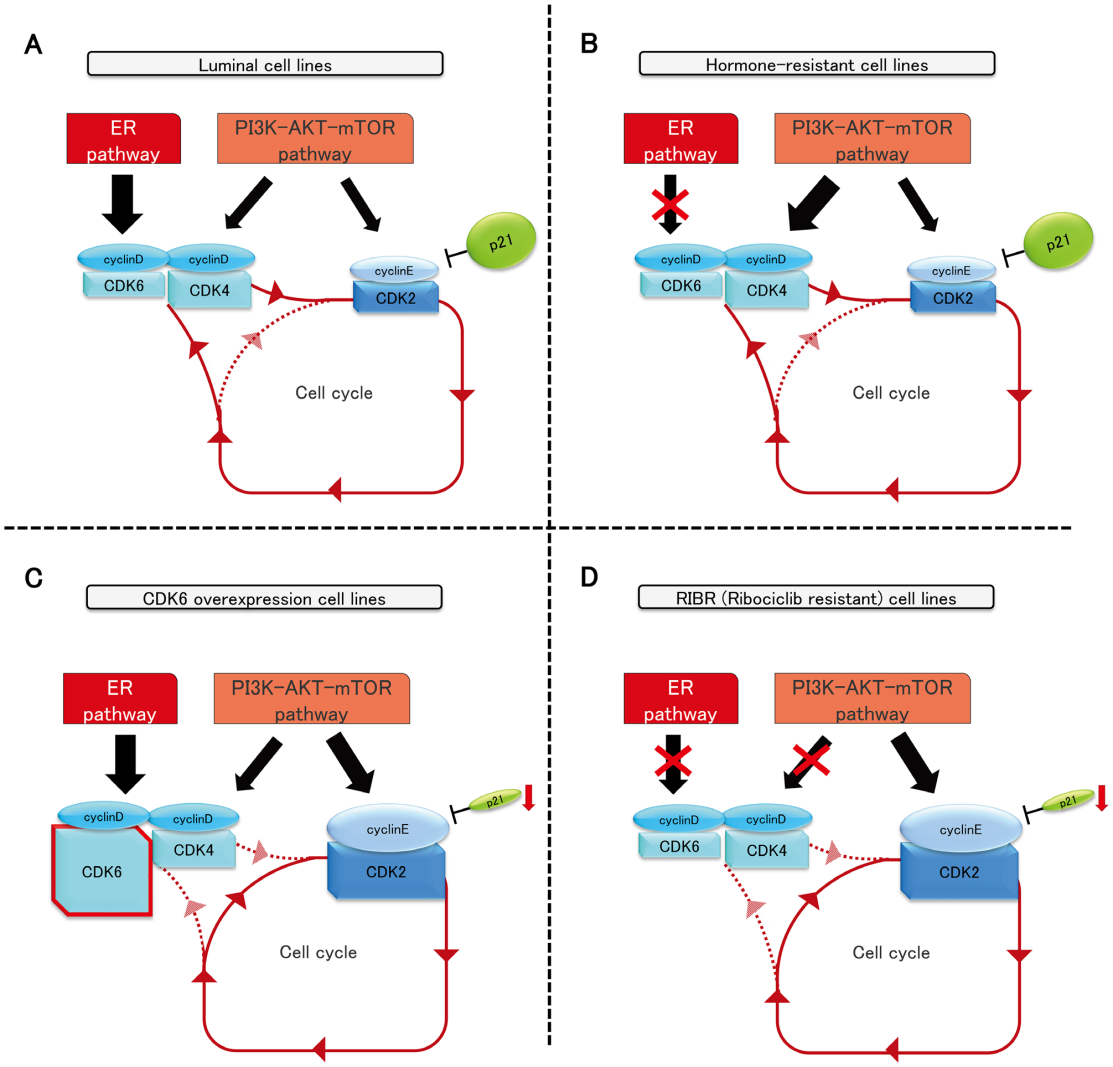
The schema of luminal, hormone-resistant, MCF7-C6, and RIBR cell lines.

As the major functional mechanism of CDK4/6 inhibitors is thought to promote late G1 stage by inhibiting S-phase immigration i.e., not cytotoxic but cytostatic activity, CDK4/6 inhibitor-resistant cells could reasonably restore the sensitivity to CDK4/6 inhibitors without the presence of these drugs after a considerable lag. Overall, p21 expression levels could play a vital role in the sensitivity to CDK4/6 inhibitors because ribociclib sensitivity was proportional to p21 expression levels. CDK4/6–cyclin D complex plays a crucial part in breast cancer, with high sensitivity to CDK4/6 inhibitors, such as luminal-type and hormone-resistant cell lines (Figure 7A and 7B). Published studies have shown emerging cyclin D1 knockout mice develop normally, but no cyclin E mutant mice were born alive, suggesting that cyclin E is a crucial target of cell-cycle progression [29, 30]. Overall, we inferred that breast cancer with low p21 expression largely depends on the CDK2–cyclin E complex to progress to the S-phase; thus, CDK4 and CDK6–cyclin D complex are no longer necessary to overcome the G1–S checkpoint (Figure 7C and 7D).

To date, effective therapies following CDK4/6 inhibitor resistance remain partially elucidated. Regarding targeted therapies, only everolimus is available for clinical use, and it has not shown promising results [31]. In the present study, we determined that all targeted therapies and chemotherapeutic drugs were effective to some extent in all CDK4/6 inhibitor-resistant cell lines. As the major functional mechanism of CDK4/6 inhibitors is considered cytostatic activity, chemotherapeutic drugs, which have cytotoxic activity, were considered effective despite resistance to CDK4/6 inhibitors. Furthermore, sensitivity to other CDK4/6 inhibitors, termed cross-resistance, was exhibited in these cell lines, not only by palbociclib but also by abemaciclib. Although pharmacological characteristics of abemaciclib are considered to be slightly different from palbociclib and ribociclib, which have a broad spectrum to CDK families [32], the degree of tolerance to abemaciclib appears similar to palbociclib. Further investigation is warranted to determine the difference of function among palbociclib, ribociclib, and abemaciclib.

In the present study, we demonstrate the multiple mechanisms of CDK4/6 inhibitor resistance and potential biomarkers of CDK4/6 inhibitors *in vitro*. Experiments on animal models are required to confirm our results. Furthermore, we should confirm the relationship between effect of these drugs and potential biomarkers through tissue and blood samples.

Our findings suggest that the mechanism of CDK4/6 inhibitor resistance exists in multiple forms and that the levels of CDK4 and CDK6 are potential biomarkers of CDK4/6 inhibitors, which may be especially useful for the selection of patients to estimate the sensitivity in breast cancer. Further, p21 could serve as a monitoring marker for CDK4/6 inhibitors. Nevertheless, further studies are warranted to elucidate the mechanism of CDK4/6 inhibitor resistance, as the findings are anticipated to provide crucial insights into considering an indication for a biomarker and monitoring marker for the resistance to CDK4/6 inhibitors.

## MATERIALS AND METHODS

### Reagents

We purchased ribociclib, palbociclib, and abemaciclib from Selleck Chemicals (Houston, TX) and obtained alpelisib from Santa Cruz Biotechnology Inc. (Santa Cruz, CA). In addition, fulvestrant was acquired from Sigma–Aldrich (St. Louis, MO), paclitaxel and U0126 were purchased from Cell Signaling Technology (Danvers, MA). Everolimus was obtained from LC laboratories, Inc. (Woburn, MA), and eribulin was acquired from Eisai (Tokyo, Japan).

In western blotting, the sources of antibodies were as follows: total Rb (#9309), pRb (Ser780; #3590), cyclin D1 (#2922), cyclin E1 (#20808), CDK2 (#2546), CDK4 (#12790), CDK6 (#13331), p21 (#2947), p27 (#2552) and β-tubulin (#2146)—all acquired from Cell Signaling Technology. In addition, we purchased horseradish peroxidase–conjugated secondary antibody from Bio-Rad Laboratories Inc. (Hercules, CA).

### Cells and cell culture

Promega provided authentication for MCF-7, T-47D, MDA-MB-231, BT20, and SKBR3 cell lines using STR-PCR. All experiments were completed within 15 passages. We stably transfected MCF7-E10 breast cancer cells derived from MCF-7 with ERE-GFR reporter plasmids as reported previously [3]. Both EDR and MFR cell lines were established from MCF7-E10 cells as described previously [3, 20]; Supplementary Figure 1 describes the character of these cells. Parent cells were maintained in RPMI-1640 medium (Sigma–Aldrich) containing 5% fetal calf serum (FCS; Gibco BRL, Grand Island, NY) and 1% penicillin/streptomycin (Gibco). In addition, MFR cell lines were maintained in fulvestrant-supplied RPMI-1640 medium (final concentration: 10 nM). In addition, two cell lines of EDR were cultured in phenol red–free RPMI-1640 medium (Gibco BRL) supplemented with 5% dextran-coated, charcoal-treated FCS and 1% penicillin/streptomycin. RIBR cell lines were established from EDR1 cell lines. We maintained RIBR cell lines in ribociclib-supplied (final concentration: 1000 nM) phenol red–free RPMI-1640 medium supplemented with 5% dextran-coated, charcoal-treated FCS and 1% penicillin/streptomycin. Furthermore, RIBR(-R) cell lines were cultured in phenol red–free RPMI-1640 medium supplemented with 5% dextran-coated, charcoal-treated FCS and 1% penicillin/streptomycin. Of note, all cells were incubated at 37°C in an atmosphere containing 5% CO_2_.

### Cell proliferation assay

In inhibitor sensitivity assays, we maintained parent, MFR, and MCF7-C6 cell lines in RPMI-1640 medium containing 5% FCS, seeded in 24-well culture plates and grown to approximately 50% confluence, added each drug for three consecutive days, harvested, and counted cells using a Sysmex CDA-1000 Automated Cell Counter (Sysmex, Kobe, Japan). EDR, RIBR, and RIBR(-R) cell lines were cultured in phenol red–free RPMI-1640 medium.

### Transfection

Using PCR, we amplified the fragment of the CDK6 coding region. The pBApo-CMV Pur DNA (TAKARA Bio Inc., Shiga, Japan) plasmid was digested separately using BamHI and HindIII restriction endonuclease. Using In-Fusion^®^ HD Cloning Kit (TAKARA Bio Inc), CDK6 fragment was ligated into the pBApo-CMV Pur DNA expression vector, which had been digested with BamHI and HindIII. We transfected the obtained CDK6 expression vector into MCF-7 using the Trans IT LT-1 Transfection Reagent (Mirus Bio LLC, Madison, WI). In addition, stable cell lines were established after selecting puromycin (1 mg/mL). Furthermore, all DNA products were confirmed by sequencing. The sequences of primers were as follows: forward, 5′-TTA GTG AAC CGG ATC CAT GGA GAA GGA CGG CCT GTG -3′ and reverse, 5′-AGC CTC CCC CAA GCT TTC AGG CTG TAT TCA GCT CCG AG-3′.

### Small interfering RNA transfection

We seeded MCF7-C6 cell lines into a 6-cm culture dish for the immunoblot analysis or 24-well culture plates for the cell proliferation assay in RPMI-1640 medium. After growing cells to approximately 50% confluence, the CDK4 or nonspecific control small interfering RNA (siRNA; concentration, 25 nM; Sigma–Aldrich) was transfected using the Lipofectamine RNAiMAX Transfection Reagent (Thermo Fisher Scientific, Waltham, MA) per the manufacturer’s instructions.

### Cell-cycle analysis

The cells were plated into a 6-cm culture dish and grown to approximately 80% confluence before adding each drug. After 24 h, cells were retrieved by trypsin treatment and fixed with 70% ethanol. The nuclei were stained with propidium iodine and analyzed on the LSR Fortessa (BD, Franklin Lakes, NJ).

### Colony formation assay

We plated 3000 cells into 6-well culture plates and replaced fresh medium and drugs every 2 or 3 days and cultured for 15 days. In addition, colonies were fixed with 4% paraformaldehyde phosphate buffer solution (Wako, Osaka, Japan) and stained with 0.3% crystal violet (Fisher Scientific, MA).

### Immunoblot analysis

We prepared cell lysates using the Lysis-M Reagent (Roche Diagnostics GmbH, Mannheim, Germany) supplemented with the PhosSTOP phosphatase inhibitor cocktail (Roche Diagnostics) per the manufacturer’s instructions. Then, extracted proteins (5 μg) were separated on 12% SDS–PAGE using acrylamide gels, and proteins were transferred to PVDF membrane. In addition, we determined protein expression by western blotting with specific antibodies listed in the reagents section and detected expression signals on the ImageQuant™ LAS 4000 Image Analyzer (GE Healthcare Bio-Sciences AB, Uppsala, Sweden) using Immun-star HRP substrate (Bio-Rad).

### Replicates

We performed three individual experiments of the cell proliferation assay and immunoblot analysis. Cell proliferation assays of CDK4 siRNA were performed in duplicate.

### Statistical analysis

We used the Student’s *t*-test to assess significance differences between two groups performed in triplicate. All data are expressed as means ± standard deviation (SD). All statistical analyses were performed using VALIDHTML. We considered *P*-values of < 0.05 as statistically significant in this study.

## SUPPLEMENTARY MATERIALS



## Abbreviations

AI: aromatase inhibitor
CDK: cyclin-dependent kinase
EDR: estrogen-deprivation resistant
ER: estrogen receptor alpha
HER2: human epidermal growth factor receptor 2
MBC: metastatic breast cancer
MFR: MCF-7-derived fulvestrant-resistant
Rb: retinoblastoma
RIBR: ribociclib-resistant cell lines
