# Supplementary material for: The p21 levels have the potential to be a monitoring marker for ribociclib in breast cancer
Source: Oncotarget. 2019 Aug 6;10(47):4907–18. doi: 10.18632/oncotarget.27127 (PMC6690670; doi:10.18632/oncotarget.27127)
Supplement: Supplementary file 1 [file oncotarget-10-4907-s001.pdf]

The p21 levels have the potential to be a monitoring marker for ribociclib in breast cancer

SUPPLEMENTARY MATERIALS

Cell lines	feature	ER expression	ER activity
MCF7—E10	Parent cell lines	+	+
EDR1	AI–resistant	++	++
EDR2		±	−
MFR	fulvestrant–resistant	−	−

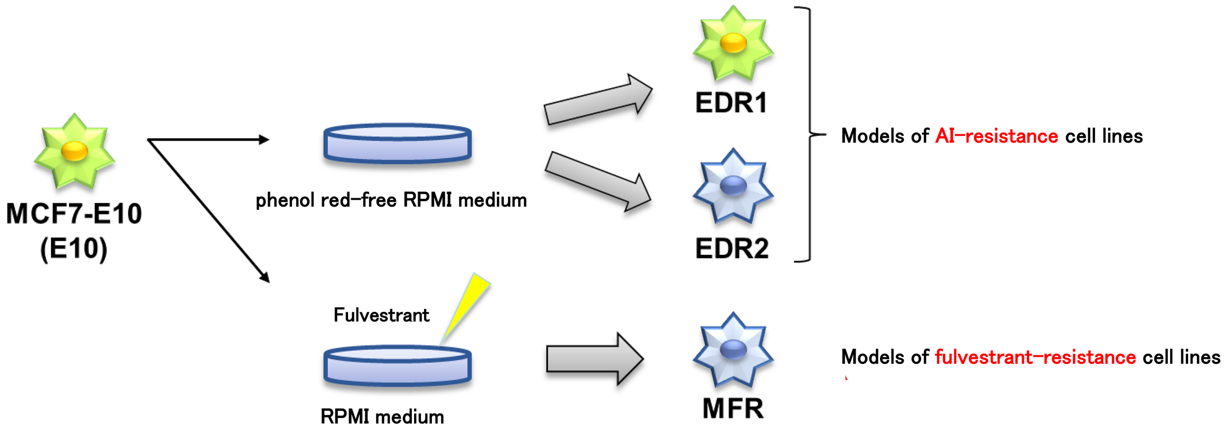

Supplementary Figure 1: Models of hormone-resistant cell lines.
